# USING ECHO TO SUPPORT DEMENTIA DIAGNOSIS IN THE PRIMARY CARE NETWORK

**DOI:** 10.1093/geroni/igz038.3274

**Published:** 2019-11-08

**Authors:** Allison Lindauer, Katherine Wild, Andrew Natonson, Miriam Wolf, Nora Mattek, Deborah Messecar

**Affiliations:** 1 Oregon Alzheimer’s Disease Research Center, Portland, Oregon, United States; 2 OHSU, Portland, Oregon, United States; 3 Oregon Health & Science University, Portland, Oregon, United States; 4 Oregon ECHO Network, Portland, Oregon, United States; 5 OHSU School of Nursing, Portland, Oregon, United States

## Abstract

Approximately 60% of those with dementia do not carry a diagnosis, undermining patient participation in clinical trials and family access to support. Under-diagnosis is driven by lack of knowledge about dementia, stigma, clinician inexperience and therapeutic nihilism. To address clinician-based contributors to under-diagnosis, we developed and implemented “Dementia 360,” a telementoring program modeled on the ECHO (Extension for Community Health Outreach) framework. Remote participants (n=67) learned about the diagnostic process, pharmacological management, family support and dementia-related resources. The video-conference-based one-hour sessions occurred weekly over 2 months. Instruction was provided by a multi-disciplinary faculty team with extensive clinical experience. Didactic presentations were followed by case studies offered by participants. Physicians, nurses, psychologists and social workers from 40 organizations participated, of which 62% were from medically underserved rural clinics. Participants were administered pre- and post-program questionnaires about their level of confidence in assessing and treating individuals with memory loss and dementia-related behavioral symptoms. Of the 54 clinicians who completed pre-intervention confidence assessments, 30 completed post-assessment. The clinicians had significantly increased confidence in diagnosing and treating dementia and managing behavioral symptoms of dementia (p ranging from .0002 to .003). Qualitative feedback from focus groups was generally positive, for example, “Knowing the diagnosis criteria and steps to take to rule out other diagnoses will help me more accurately diagnose and rule out dementia for my patients.” Our findings suggest that delivering case-based education via ECHO has potential to increase clinician workforce confidence in diagnosing and managing dementia.

